# Characteristics of Submucous Myomas and the Risk of Anemia

**DOI:** 10.3390/medicina58111652

**Published:** 2022-11-15

**Authors:** Giuseppe Ricci, Federica Scrimin, Andrea Sartore, Massimo Borelli, Gabriella Zito, Federico Romano, Guglielmo Stabile

**Affiliations:** 1 Institute for Maternal and Child Health, IRCCS Burlo Garofolo, 34137 Trieste, Italy; 2 Department of Medicine, Surgery and Health Sciences, University of Trieste, 34127 Trieste, Italy

**Keywords:** myomas, anemia, metrorrhagia, myoma grade, hemoglobin

## Abstract

*Background and Objectives*: Uterine fibroids still represent the most common indication for hysterectomy for benign pathologies. In the United States, more than 479,000 hysterectomies are performed annually, 46.6% for myomas and 47.7% in women aged from 18 to 44 years. By applying appropriateness criteria to this procedure, it has been estimated that overuse ranges from 16 to 70%. One of the main reasons that induce patients and gynecologists to consider hysterectomy is represented by severe anemia. *Materials and Methods*: This is a retrospective cohort study of 202 patients with uterine fibroids diagnosed by transvaginal ultrasound who underwent a hysteroscopic procedure. Myoma grade, size, location, and number were assessed by transvaginal scan and office hysteroscopy and correlated to the pre-treatment hemoglobin level. *Results*: Univariate analysis showed that anemia does not have a statistically significant association with myoma number and with age considered as a numerical predictor. In the patients with myoma type 0, there is a possibility of 81% having anemia regardless of menorrhagia. On the contrary, in patients with myoma type 1 or type 2, the possibility of having anemia varies according to the presence or absence of menorrhagia. If there is menorrhagia, the risk of moderate anemia is only present for myomas >60 mm. *Conclusions*: The results of this study may contribute to defining objective criteria for the management of submucous myomas and anemia. Our data suggest that submucosal myomas type 0 >10 mm should always be treated, putting patients at risk for anemia. Myomas type 2 and 3 should be treated for the risk of anemia in the presence of menorrhagia episodes or if > of 60 mm. Adequate management of anemia and myomas could reduce the rate of unnecessary hysterectomies.

## 1. Introduction

In the United States, more than 479,000 hysterectomies are performed annually, 46.6% for myomas and 47.7% in women aged from 18 to 44 years, although they have been introduced to uterine sparing surgical options [1]. These data raise a problem considering that two-thirds of patients using assisted reproduction techniques are older than 34 years of age [2], and the average age of women seeking out fertility treatments, in general, has been steadily increasing, as happens for the prevalence of myomas in the population after 35 years (up to 40%) [3]. However, there is a wide variation in rates of hysterectomy. By applying appropriateness criteria to this procedure, it has been estimated that overuse ranges from 16 to 70% [4]. The main reason for inappropriateness was the lack of precise diagnostic and therapeutic intervention before surgery [5,6].

Myoma management guidelines address treatment strategies on symptoms, age, desire for future pregnancies, and proximity to menopause [7,8,9,10]. However, there is no clear definition of the myoma features requiring surgery [11,12]. One of the main reasons that induce patients and gynecologists to consider hysterectomy is represented by severe anemia [13,14]. This is due to the fact that severe anemia, in addition to representing a serious health problem for the patient, may suggest that the uterine mass may be malignant. Several studies investigated the correlation between submucous myoma characteristics and anemia with conflicting results [15,16,17,18,19,20,21,22].

Nonetheless, only in two studies submucous myomas were diagnosed by hysteroscopy, and objective criteria to define anemia were used [20,21]. It has been demonstrated that hysteroscopy is superior to other diagnostic techniques in evaluating submucous myomas and that self-reported bleeding scores are not as effective as hemoglobin for identifying anemia. However, the first study evaluated only the grade of myoma, and the second investigation included only women with a single myoma.

At a biological level, anemia develops because of an imbalance in erythrocyte loss relative to production; this can be due to ineffective or deficient erythropoiesis (e.g., from nutritional deficiencies, inflammation, or genetic hemoglobin disorders) and/or excessive loss of erythrocytes (due to hemolysis, blood loss, or both). Defining an abnormally low hemoglobin (Hb) concentration requires understanding how Hb naturally varies by age, sex, pregnancy status, genetic and environmental factors, and, potentially, race [23]. The present study examines the relationship between several myoma characteristics and anemia in women with multiple myomas. Early identification of women at risk of anemia could lead to early medical and possibly surgical therapy instead reducing surgery to women considered at low risk of anemia.

## 2. Materials and Methods

This retrospective cohort study included premenopausal women with multiple myomas, with at least one submucous myoma, diagnosed by transvaginal ultrasound, who underwent hysteroscopic procedures at the Gynecology Clinic of the Institute for Maternal and Child Health, IRCCS Burlo Garofolo, Trieste, Italy. Exclusion criteria were menopause, coagulopathies (von Willebrand disease, thrombocytopenia), medical disorders (hepatic failure, gastritis, malabsorption, disorders of iron metabolism), use of anticoagulants, antifibrinolytic agents, nonsteroidal anti-inflammatory drugs, current hormone treatments. Patients with a Body Mass index <17 were excluded from our study to avoid eating disorders as a confounding factor for anemia. Furthermore, patients taking iron therapy were automatically excluded from the study. Hysteroscopy and endometrial biopsy were performed within 30 days after the ultrasound. Women with polyps, endometrial hyperplasia, or cancer were excluded from the study.

Menorrhagia was determined subjectively by direct questioning. Hemoglobin level was determined at the first visit before hysteroscopy and any medical treatment (iron supplementation, GnRH analogs, blood transfusion). Anemia severity was categorized into three groups: mild (10.1–12 g/dL), moderate (8.1–10 g/dL), and severe (≤8 g/dL) [24].

A transvaginal ultrasound scan was used to assess myomas’ number, position (intramural, subserous, submucous), size, and location (fundus, corpus, isthmus). The sonographic equipment consisted of a Voluson E8 (GE Healthcare, Zipf, Tiefenbach, Austria). Two qualified physicians performed the ultrasound measurement according to a standardized protocol: the uterus was visualized in both sagittal and coronal planes to determine the presence or absence of myomas [25]. The length, height, and width of the largest myoma were measured when multiple myomas were detected.

The office hysteroscopy was conducted after ultrasound evaluation with Bettocchi Hysteroscope 5.0 mm continuous flow (Karl Storz- Endoskope, Tuttlingen, Germany) by two experienced physicians according to standard procedure, without anesthesia [26].

Submucous myomas were classified according to the European Society for Gynecological Endoscopy (ESGE) classification, which defines the proportion of myoma in the uterine cavity [27]. Type 0 refers to myomas entirely located in the uterine cavity, type 1 refers to myomas with more than 50% of their volume protruding into the uterine cavity, and type 2 with less than 50% of myoma volume protruding into the uterine cavity.

The study was approved by the Institutional Review Board of IRCCS Burlo Garofolo, Trieste, Italy (IRB 08/2020, 15 April 2020).

### Statistical Analysis

Descriptive statistics of numerical data were provided by median and range; categorical data were expressed by number and relative frequencies. To evaluate the association between the anemia risk and myoma grade, location, size, and menorrhagia, exploratory multivariable analyses were conducted with regression trees [28]. Within exploratory analyses, myoma size was considered both a categorical and continuous variable. Univariate analyses over 2 by 2 contingency tables were addressed by Fisher exact test and summarized by odds ratio, while on continuous covariate, the logistic regression was addressed. Multivariable inferential analyses were performed with linear models or, accordingly, generalized linear models (logistic regressions), and model selection was achieved by Akaike Information Criteria, according to a top-down method and correcting for overdispersion [29,30]. In all inferential instances, an alpha level = 0.05 was assumed, and the statistical analysis was carried out with the statistical package R 3.4.0 [31].

## 3. Results

Two hundred and two patients who underwent a hysteroscopy were included in this study. The number of participants with missing data in the variables studied was six. Table 1 and Table 2 show the characteristics of the study population. In our population, 73 patients were found with mild anemia (36.13%), 40 women were found with moderate anemia (19.8%), and 14 with severe anemia (6.93%). In general, 127 women were anemic (62.87%).

Univariate analyses disclosed that anemia binomial response (no/yes, according to a 12 g/dL value cut-off) does not have a statistically significant association with myoma number (Fisher test, *p*-value = 0.54, odds ratio = 0.81) and with age considered as a numerical predictor (logistic regression, *p*-value = 0.20). Multivariate logistic regression analysis was employed to study the association among other potential predictor variables: grade (type 0, 1, or 2), size, location, and menorrhagia. A minimal adequate model involving only grade and menorrhagia in a significant statistical sense was detected, performing a top-down selection within multivariable logistic models through lower Akaike Information Criteria (AIC), allowing for interaction between covariates and correcting—when necessary—for overdispersion of the residuals, as reported in Table 3.

**Figure 1:**
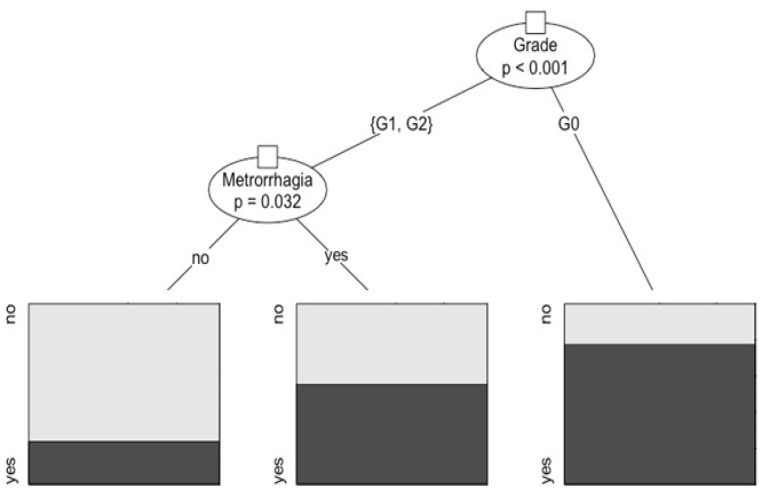
The regression tree depicting the relation between anemia and its statistically significant predictors, i.e., grade and menorrhagia/metrorrhagia. In the patients with myoma type 0, there is a possibility of 81% having anemia regardless of menorrhagia/metrorrhagia. In the patients with myomas type 1–2, the possibility of having anemia varies according to the presence or absence of menorrhagia: in the former case, the possibility is estimated at 54%, while in the latter case, the possibility decreases to 24%.

Moreover, our exploratory analysis led through the regression trees technique discloses that in a patient with a myoma type 0, there is a possibility of 81% having anemia regardless of menorrhagia/metrorrhagia, which does not alter such probability in a statistically significant manner. On the contrary, in the patients with myoma type 1 or type 2, the possibility of anemia varies according to whether or not they have menorrhagia: in the first case, the possibility is estimated at 54%, while in the second, the chance decreases by 24% (Table 4, Figure 1).

We also investigated the association between hemoglobin levels and study covariates. The minimal adequate models revealed a more complex structure, with significant interaction with myoma size, myoma grade, and menorrhagia (Table 5 and Table 6).

Table 7 summarizes hemoglobin level variations according to our statistical model, depending on predictors: in the patients with submucous myoma, hemoglobin level decreases with more extensive myoma size, and the decrease is higher in the case of patients with menorrhagia.

Moreover, the model shows that patients with myoma type 0 >10 mm are ever at risk of anemia, mild or moderate. In these patients, if there is no menorrhagia, the risk of moderate anemia is significant only for myomas >60 mm; if there is menorrhagia, the risk of moderate anemia for myomas >30 mm increases.

Patients with type 1–2 myomas without menorrhagia do not have a significant risk of moderate anemia. In the presence of menorrhagia, the risk of moderate anemia is present only for myomas > 60 mm.

**Table 7:** Interaction among hemoglobin level, myoma grade, myoma size, and menorrhagia.

Myoma Grade	Menorrhagia	Myoma Size (mm)						
		0–10	10–20	20–30	30–40	40–50	50–60	60–70
**Type 0**	**No**	12.36	11.91	11.46	11.01	10.56	10.11	<=9.66
	**Yes**	11.34	10.89	10.44	9.99	9.54	9.09	<=8.64
**Type 1 or 2**	**No**	13.51	13.06	12.61	12.16	11.71	11.26	<=10.81
	**Yes**	12.49	12.04	11.59	11.14	10.69	10.24	<=9.79

Green: no anemia; yellow: Mild anemia; orange: Moderate anemia.

## 4. Discussion

We found that the only significant risk factors for anemia were myoma protrusion grade and menorrhagia. Moreover, based on our statistical analysis, patients with submucous myoma can be grouped into subjects with type 0 and individuals with type 1–2 myomas.

In patients with type 0 myoma, there is a possibility of 81% having anemia. Menorrhagia does not influence such probability in a statistically significant manner. In patients with type 1–2 myomas, the risk of anemia is lower and depends on the presence of menorrhagia. This difference is most likely because, in patients with type 0 myomas, the duration and amount of menstrual bleeding are more significant than in patients with type 1–type 2 myomas. Many factors can explain the association between type 0 myomas and heavy menstrual bleeding [32,33].

Our results partially agree with the study by Puri et al. [21], in which the risk of anemia was not significantly increased in patients with class 1 myoma (more than 50% in the cavity). However, they did not consider the size of the myoma and menorrhagic period.

In our research, we preferred to study women with multiple myomas, as up to 84% of the female population with uterine fibromatosis have multiple myomas [34].

Our findings confirm a more complex relationship between myoma features and Hb in women with multiple myomas, as observed by Yang et al. [20] in subjects with single myoma.

These studies did not consider the location and number of myomas [20,21]. Using a reliable diagnostic method and strict criteria for analyzing submucous myoma and anemia, respectively, we showed that neither location (uterine fundus, isthmus, or corpus) nor the number of myomas played a significant role in the risk of developing anemia. Therefore, our study adds important information to knowledge on this topic.

To provide a practical tool to support counseling for patients with submucous myoma, we further analyzed the interaction between the significant variables. Thus, we developed a simple mathematical model to predict hemoglobin values using our population data. This model considers only three variables: grade, size of myoma, and menorrhagia (yes/no).

This model shows that patients with myoma type 0 >10 mm are always at risk of mild or moderate anemia. The threat of moderate anemia is relevant only for myomas >60 mm if there is no menorrhagia. If there is menorrhagia, the chance of moderate anemia is present for myomas >30 mm. Patients with type 1–type 2 myomas without menorrhagia are not significantly at risk of moderate anemia. If there is menorrhagia, the risk of moderate anemia is only present for myomas >60 mm.

Considering that we investigated a large population and applied an effective statistical method, this model could be used in the surgical decision-making process.

Uterine myomas are the most common benign gynecological tumors and are present in 30% of women of reproductive age [35]. Despite the frequency, there is uncertainty and controversy among clinicians and women regarding the best treatment modality for myomas [36,37,38].

The treatment choice is often influenced by the attitude of the gynecologist, his surgical experience, the work environment, and the patient’s preferences [36]. One of the main reasons that induce patients and gynecologists to consider hysterectomy is represented by severe anemia [13,14].

Therefore, it is fundamental to identify the more relevant factors that influence hemoglobin levels in women with myomas to provide objective criteria for establishing the appropriateness of surgery. Several studies have investigated the correlation between menstrual bleeding patterns and myoma characteristics with conflicting results [15,16,17,18,19,20,21,22].

Two studies conducted in the non-care-seeking population in USA and Italy concluded that there is no relationship between submucous myoma, diagnosed using ultrasound, and heavy menstrual bleeding. The American study found that heavy bleeding increases with myoma size, but the presence of submucous myoma or multiple myomas does not influence the risk of gushing-type bleeding after adjusting for the size of the most extensive myoma [15]. The results of the Italian study showed that the number, volume, tissue layer location (subserosal, intramural, or submucous), and axial position (anterior or posterior) of the myoma were not related to menstrual cycle characteristics [16]. However, this conclusion was based only on three cases of submucous myomas. Both studies evaluated the characteristics of menstrual bleeding on self-reported data obtained from interviews.

Bachman et al. [19] evaluated symptomatic women with myomas using ultrasound in symptomatic women. They found that symptomatic women with at least one submucous myoma are not at higher risk of becoming anemic than symptomatic women with non-submucous myomas. An epidemiological study in Germany obtained similar results [22]. The number and size of myomas, as determined by ultrasound, were significant risk factors for the presence of heavy menstrual bleeding, but there was no link between myoma location and heavy menstrual bleeding [22]. Two other studies using MRI showed that the size and location of myomas, as determined by MRI, were not related to menstrual characteristics [17,18]. However, in the study by Sulaiman et al. [17], all the women with sub-mucosal myomas had menorrhagia with a menstrual blood loss higher than 80 mL.

In two studies, submucous myomas were detected by ultrasound, hysteroscopy, and objective criteria to define anemia were used [20,21]. Hysteroscopy has been shown to be superior to other diagnostic techniques in assessing submucous myoma, and self-reported bleeding scores are not as effective as hemoglobin for identifying anemia.

Puri et al. [21] observed that hemoglobin was negatively associated with class 0 myoma and class 1 myoma, according to ESGE classification, compared to women without submucous myoma. Similarly, the risk of anemia was increased with class 0 myoma and non-significantly with class 1 myoma. However, they did not consider the size of the myoma and menorrhagic period.

**Figure 2:**
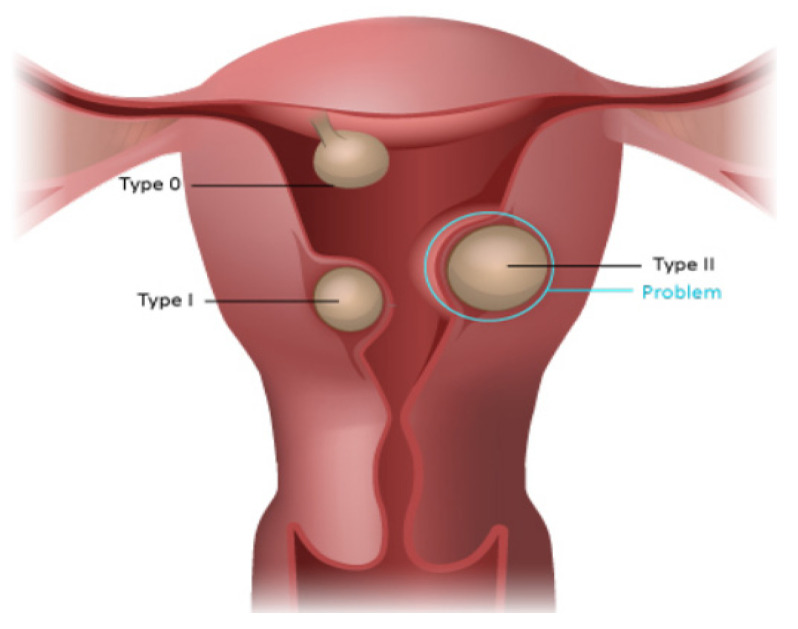
ESGE classification of submucous myomas. They classified the submucous myomas into three groups according to their protruding %: <50%, 50–79%, and >80%. From: (https://elearning.rcog.org.uk//uterine-cavity-surgery/hysteroscopic-myomectomy/classification-submucous, accessed on 30 August 2022).

Yang et al. [20] investigated women with single submucous myoma and used a mildly different classification compared to the ESGE classification (Figure 2). They classified the submucous myomas into three groups according to their protruding %: <50%, 50–79%, and >80%. They reported that as the protruding % increased, there was a significant trend toward a higher risk of moderate to severe anemia. This chance increased after an adjustment for myoma size and menorrhagia period.

By using the same methodology, our study investigated women with multiple myomas evaluating several myoma features.

Our analysis suggests that a myoma type 0 over 10 mm should be ever treated, considering that in this case, there is always a risk of mild or moderate anemia. Myomas type 2 and 3 should be treated in the presence of menorrhagia episodes, considering the risk of moderate anemia only in this case or if the myoma size is over 60 mm.

Our study has several strengths. Consecutive patients were included to obtain good generalizability of the results. The study population was not selected based on socio-economic status, education, and race, as our hospital is the only one in the city.

Hysteroscopic and ultrasound procedures were performed by a small group of highly qualified physicians (two for hysteroscopy and two for ultrasound), and the same instrumentation was used for all the patients. Therefore, potential biases in the evaluation of myoma characteristics were negligible. Finally, an effective statistical method was adopted.

There are also some limitations of this study. The main limitation is its retrospective nature. Our sample is limited (202 patients), and it is a biased sample considering that the study included only women who attended a gynecological consultation. Data were not collected prospectively, but we conducted a rigorous systematic chart review. The patient’s clinical record contained all the necessary information to carry out statistical analyses. Another limitation of the study is the subjective definition of menorrhagia. No objective method of assessing menstrual blood loss was employed, but menorrhagia was determined subjectively by direct questioning. Furthermore, recent weight change, body mass index, the presence of regular or irregular menses, and of adenomyosis or hyperplasia were not considered.

To the best of our knowledge, this is the first study that evaluates the association between myoma characteristics and anemia in women with multiple myomas.

## 5. Conclusions

In some cases, myoma therapy is based on the physician’s subjective criteria. The results of this study may contribute to defining objective criteria for managing submucous myomas and anemia. The analysis of myoma grade, myoma size, and the presence of menorrhagia could lead to early detection of women with anemia. Furthermore, our data suggest that submucosal myomas type 0 >10 mm should always be treated, putting patients at risk for anemia. Myomas type 2 and 3 should be treated for the risk of anemia in the presence of menorrhagia episodes or if > of 60 mm. This observation (with the absence of ultrasound risk characteristics, normal markers, and myoma not rapidly growing) could lead to a reduction in surgical therapy of these types of myoma and perhaps a general reduction in the number of hysterectomies, but further studies are needed.

Furthermore, adequate management of anemia with early medical therapy or a minimally invasive surgical approach (hysteroscopy) that can only lead to the removal of submucosal myomas could reduce the rate of unnecessary hysterectomies.

## Figures and Tables

**Table 1:** Study population characteristics.

Characteristics	*n* = 202
**Age, year**	44.1 (32–55)
**Myoma size, mm**	30.0 (5–70)
**Myoma location**	
**Fundus**	78 (38.6)
**Corpus**	36 (17.8)
**Isthmus**	19 (9.4)
**Other**	69 (34.2)
**Hb at the first visit, g/dL**	10.6 (3.3–14.5)

Values are median (minimum-maximum) or *n* (%).

**Table 2:** Patients with anemia.

**Total patients with anemia**	127 (62.87%)
**Mild anemia**	73 (36.13%)
**Moderate anemia**	40 (19.8%)
**Severe anemia**	14 (6.93%)

**Table 3:** The univariate analysis.

Covariate	*p*	AIC
Metrorrhagia	<0.01	173.1
Grade	0.01	238.9
Histmic	0.09	240.4
Dimensions	0.01	242.9
Number	0.24	254.8
Age	0.44	256.4

**Table 4:** Probability of anemia related to myoma grade and menorrhagia.

	Myoma Grade	Probability
	**Type 0**	81%
	Menorrhagia/metrorrhagia	
Type 1 or type 2	Yes	54%
Type 1 or type 2	No	24%

**Table 5:** Multivariate logistic regression analysis among predictor variables associated with anemia.

	Estimate	Standard Error	*p*-Value
**Intercept**	0.39	0.37	0.29
**Type 1 or type 2**	−1.45	0.38	<0.001
**Menorrhagia**	1.06	0.40	0.01

**Table 6:** Association among hemoglobin levels and study covariates.

	Estimate	Standard Error	Pr(>|t|)
**Intercept**	15.44	2.47	<0.001
**Type 1 or type 2**	1.24	0.31	<0.001
**Menorrhagia**	−1.27	0.34	<0.001
**Size**	−0.19	0.07	0.01

## Data Availability

The original contributions presented in the study are included in the article; further inquiries can be directed to the corresponding author.
